# Light‐Inducible Activation of FGFR3 Facilitates Chondrocyte Maturation

**DOI:** 10.1111/cpr.70218

**Published:** 2026-05-04

**Authors:** Mengze Sun, Yun Zhao, Yuqing Du, Yifei Fan, Kai Wang, Xiaoqing Hu

**Affiliations:** ^1^ Department of Sports Medicine, Institute of Sports Medicine of Peking University, Beijing Key Laboratory of Sports Injuries Peking University Third Hospital Beijing China; ^2^ Department of Physiology and Pathophysiology, School of Basic Medical Sciences, State Key Laboratory of Vascular Homeostasis and Remodeling Beijing Advanced Center of Cellular Homeostasis and Aging‐Related Diseases, Clinical Stem Cell Research Center, Peking University Third Hospital, Peking University Beijing China

## Abstract

Light‐inducible activation of FGFR3 induced robust activation of MAPK signaling, promoting proliferation and collagen depositon in induced chondrocytes and prevent the degeneration of osteoarthritic chondrocytes.

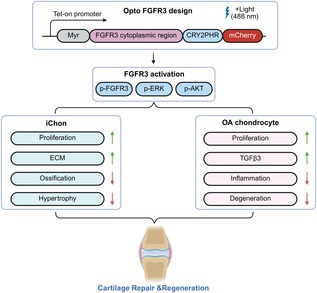

To the Editor,

1

Cartilage defects arising from trauma, inflammatory conditions or degenerative diseases affect nearly one in five adults and remain a major cause of chronic pain and disability [1]. Despite advances in biomaterials and surgical strategies, effective restoration of hyaline cartilage and prevention of osteoarthritis (OA) progression remain unmet clinical needs due to the intrinsic avascularity and limited regenerative capacity of articular cartilage [2]. Current interventions, including microfracture and pharmacological treatments, often result in fibrocartilage formation or progressive degeneration [3]. To date, human pluripotent stem cell (hPSC)‐based therapy represents a promising alternative. However, induced chondrocytes (iChons) frequently exhibit insufficient type II collagen (COLII) secretion and a strong tendency toward hypertrophy and endochondral ossification after transplantation [4]. These deficiencies highlight the functional immaturity of iChons and impede the clinical translation of hPSC‐based therapy.

To promote functional maturation of iChons, we sought to modulate fibroblast growth factor (FGF) signalling, a pathway essential for chondrocyte development, matrix homeostasis and growth regulation [5]. Among the FGF receptors, FGFR3 exhibits a distinct expression pattern, being present in proliferative and prehypertrophic chondrocytes but largely absent in hypertrophic cells, and is required for extracellular matrix synthesis and balanced proliferation. Notably, FGFR3 expression is reduced in osteoarthritic cartilage, and exogenous delivery of FGF18 mRNA has been shown to alleviate disease progression via activation of FGFR3 signalling, supporting its disease‐modifying role [6]. These observations suggest that precise activation of FGFR3 signalling may enhance chondrocyte maturation while preventing premature hypertrophy, thereby offering a promising strategy for improving the functional quality of hPSC‐derived chondrocytes.

To precisely control FGFR3 activation, we engineered a blue‐light‐responsive FGFR3 construct (OptoFGFR3) by fusing the cytoplasmic domain of FGFR3 (FGFR3c) to the photoreceptor homodimerization domain of cryptochrome‐2 (CRY2PHR) and an mCherry reporter (Figure 1A, see Materials and Methods in the Supporting Information for full details). We hypothesised that light‐induced clustering of CRY2PHR would spatially approximate FGFR3c and thereby initiate receptor signalling independent of ligand binding. Although an N‐terminal myristoylation signal peptide was introduced to facilitate membrane localization, OptoFGFR3 displayed both plasma membrane and nuclear distribution in HEK293T cells (Figure 1B). Upon 488‐nm illumination, nuclear‐localised OptoFGFR3 rapidly underwent phase‐like condensation along the nuclear membrane, accompanied by a reduction in nuclear mCherry fluorescence (Figures 1C and S1A). The localization of OptoFGFR3 on nuclear membrane was further validated using nuclear staining (Figure S1B). Notably, the phase separation of OptoFGFR3 occurred within seconds (Video S1). To test whether downstream signalling was activated, we quantified phosphorylation of FGFR3, ERK and AKT (T308). Blue‐light stimulation combined with doxycycline induction markedly increased phosphorylation of FGFR3 and ERK, and elevated AKT phosphorylation (Figure 1D,E). Consistently, p‐ERK immunostaining revealed a higher frequency of p‐ERK^+^ cells upon OptoFGFR3 activation (Figure 1F,G). Together, these observations demonstrate that OptoFGFR3 enables rapid and ligand‐independent activation of FGFR3 downstream signalling in response to blue‐light stimulation.

**FIGURE 1:**
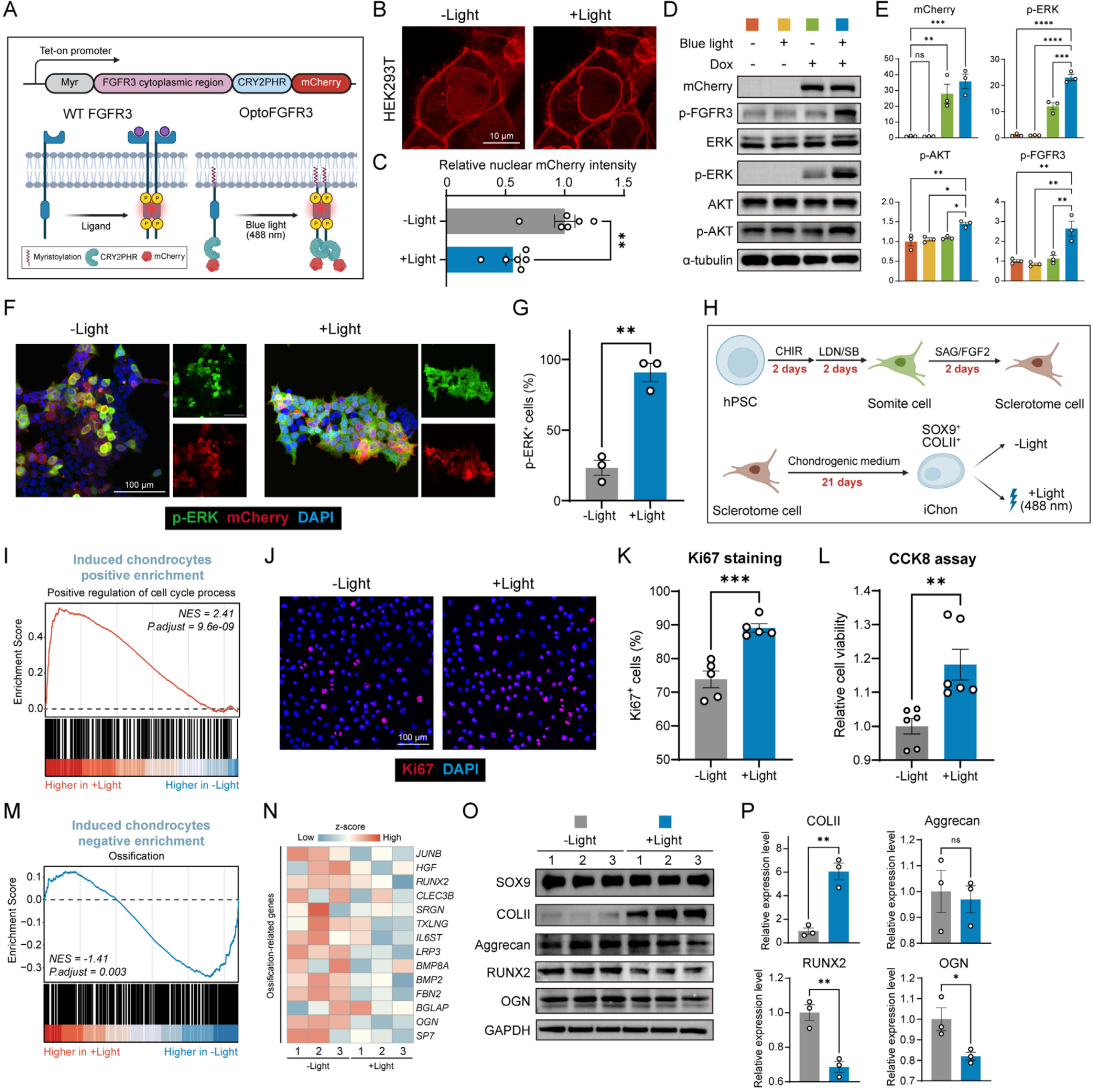
Optogenetic activation of FGFR3 promotes chondrocyte maturation. (A) Schematic illustration demonstrating the design of the OptoFGFR3 construct. (B) Representative confocal images of OptoFGFR3‐HEK293T cells before and after illumination. Scale bar: 10 μm. (C) Quantification of the nuclear mCherry intensity before and after illumination. (D) Western blot analysis of phosphorylation of ERK, AKT and FGFR3. (E) Quantification of phosphorylation levels of ERK, AKT and FGFR3. (F) Immunostaining of p‐ERK in OptoFGFR3‐HEK293T cells. Scale bar: 100 μm. (G) Quantification of the p‐ERK staining. (H) Schematic illustration demonstrating the chondrogenic differentiation of OptoFGFR3‐engineered stem cells to induce chondrocytes (iChons) and light‐mediated FGFR3 activation. (I) Gene‐set enrichment analysis (GSEA) of cell cycle‐related pathway. (J) Immunostaining of Ki67 in OptoFGFR3‐iChons. Scale bar: 100 μm. (K) Quantification of Ki67‐positive cell fraction. (L) CCK8 assay of OptoFGFR3‐iChons. (M) GESA of ossification pathway. (N) Heatmap showing expression patterns of ossification‐associated genes. (O) Western blot analysis on the protein levels of SOX9, Aggrecan, type II collagen, RUNX2 and OGN in iChons. (P) Quantification of protein levels of Aggrecan, type II collagen, RUNX2 and OGN.

To investigate the biological function of OptoFGFR3, we established a dox‐inducible OptoFGFR3 H9 hPSC line, enabling temporally controlled expression specifically after chondrogenic differentiation (Figure 1H). Engineered hPSCs remained in expression of pluripotency‐related markers OCT4, NANOG and SOX2 (Figure S1C). And the hPSCs robustly expressed OptoFGFR3 after adding doxycycline (Figure S1D). Then the OptoFGFR3 hPSCs were sequentially induced toward somite and sclerotome lineages, followed by three‐dimensional pellet culture to generate iChons. Because of limited optical penetration of blue light in compact pellets, cells were dissociated prior to downstream analyses. Quantitative polymerase chain reaction (qPCR) analysis revealed expression of somite markers *MEOX1, PAX3* and *TCF15* in induced somite cells and expression of sclerotome markers *PAX9* and *NKX3‐2* in induced sclerotome cells, indicating successful lineage specification (Figure S1E,F and Table S1). Immunostaining confirmed successful chondrogenic differentiation with robust expression of SOX9, COLII and ACAN before and after dissociation (Figure S1G,H). RNA sequencing (RNA‐seq) profiling and principal component analysis (PCA) revealed clear separation between light‐activated and non‐activated samples, indicating robust transcriptional rewiring driven by OptoFGFR3 activation (Figure S1I). Pathway enrichment revealed that light activation prominently enriched gene signatures related to cell cycle progression (Figure 1I), indicating proliferative stimulation by OptoFGFR3. Additionally, both Ki67 staining and CCK‐8 assay demonstrated enhanced proliferation upon light stimulation (Figure 1J–L), consistent with the reported effect of FGF18 in promoting chondrocyte expansion [6]. Importantly, gene‐set enrichment analysis (GSEA) further showed negative enrichment of ossification pathways (Figure 1M), and transcript levels of multiple osteogenic regulators, including *HGF*, *RUNX2*, *BMP2*, *FBN2*, *OGN* and *SP7*, were significantly suppressed upon OptoFGFR3 activation (Figure 1N). Protein analyses corroborated these findings, showing reduced levels of RUNX2 and OGN, together with a marked increase in COLII deposition, whereas Aggrecan levels remained unchanged (Figures 1O,P and S1G). Collectively, these results demonstrate that precise, optically controlled activation of FGFR3 signalling not only promotes iChon proliferation but also enhances ECM synthesis while attenuating premature ossification. Manipulating FGFR3 signalling via OptoFGFR3 induced a more mature phenotype of iChons.

To further evaluate the translational potential of OptoFGFR3, we transduced human primary OA chondrocytes with Lenti‐OptoFGFR3 and performed transcriptomic profiling (Figure S2A). PCA also revealed clear separation between light‐activated and non‐activated samples (Figure S2B). Upon blue‐light activation, GSEA revealed strong enrichment of cell‐cycle‐associated pathways, indicating enhanced proliferative capacity in primary chondrocytes (Figure S2C). In parallel, negatively enriched pathways were largely associated with inflammation, suggesting that OptoFGFR3 activation may attenuate degenerative signalling cascades characteristic of OA progression (Figure S2D). Moreover, transcriptional upregulation of *TGFB3*, a key paracrine regulator that reinforces articular chondrocyte identity, was observed [7], accompanied by reduced expression of fibrocartilage‐associated markers *FN1* and *FBN1* (Figure S2E). Collectively, these findings demonstrate that OptoFGFR3 not only preserves articular chondrocyte identity but also counteracts pathological progress of OA chondrocytes, extending the applicability of this optogenetic system toward potential therapeutic modulation of diseased human cartilage tissues.

In conclusion, this work establishes OptoFGFR3 as a highly precise and modular strategy for interrogating and restoring FGFR3 signalling in both stem cell‐derived and diseased human chondrocytes. Unlike conventional ligand‐based activation, the optogenetic design enables rapid and ligand‐independent stimulation of FGFR3, resulting in enhanced proliferation, improved COLII deposition and suppression of hypertrophic and ossification programmes. Importantly, the ability of OptoFGFR3 to reprogram primary OA chondrocytes toward a less degenerative transcriptional state highlights its translational relevance. However, the limited penetration of blue light in OptoFGFR3 may restrict its translational potential. Future efforts will focus on developing and utilising far‐red optogenetic systems to enable deep‐tissue FGFR3 activation and promote in vivo therapeutic applications for OA. As a result, our research provides mechanistic insight into how temporally controllable FGFR3 activation influences chondrocyte maturation and maintenance, and introduces an optogenetic platform that could be broadly applied to dissect signalling dynamics during cartilage development, degeneration and repair. Thus, OptoFGFR3 represents an innovative biological tool with potential to guide the optimization of hPSC‐based cartilage regeneration strategies and to inform targeted interventions for OA.

## Author Contributions

Mengze Sun and Yun Zhao conducted experiments and wrote the original draft. Yuqing Du and Yifei Fan conducted experiments. Xiaoqing Hu and Kai Wang conceptualised and supervised the study and acquired funding.

## Funding

This work was supported by the National Key R&D Program of China (2025YFC3408802), National Natural Science Foundation of China (U23A6009, 8247091728), and Peking University Medicine plus X Pilot Program‐Platform Construction Project (2024YXXLHPT009).

## Conflicts of Interest

The authors declare no conflicts of interest.

## Supporting information


**Figure S1:** Construction and validation of OptoFGFR3 cell line. (A) Confocal images of OptoFGFR3‐HEK293T cells before and after illumination with blue laser. Scale bar: 10 μm. (B) Confocal images of OptoFGFR3‐HEK293T cells after illumination with blue laser, counterstained with DAPI. Scale bar: 10 μm. (C) Immunostaining of pluripotency markers on OptoFGFR3 hPSCs. Scale bar: 200 μm. (D) Fluorescent image of OptoFGFR3 hPSCs after adding doxycycline. (E) qPCR analysis of marker genes expression of somite cells. (F) qPCR analysis of marker genes expression of sclerotome cells. (G) Immunostaining of type II collagen (COLII) and ACAN on chondrocyte pellets. Scale bar: 200 μm (left panel) and 100 μm (right panel). (H) Immunostaining of SOX9 and COLII on induced chondrocytes. Scale bar: 50 μm (left panel) and 100 μm (right panel). (I) Principal component analysis of RNA‐seq data from induced chondrocytes with or without blue light illumination.
**Figure S2:** OptoFGFR3 inhibits the degeneration of human primary osteoarthritic chondrocytes. (A) Schematic illustration demonstrating the isolation of human primary chondrocytes and lentivirus transduction. (B) Principal component analysis of RNA‐seq data from primary chondrocytes with or without blue light illumination. (C) Gene‐set enrichment analysis (GSEA) on cell cycle‐related pathway. (D) GSEA on inflammation‐related pathway. (E) RNA‐seq expression levels of *TGFB3*, *FN1* and *FBN1*.
**Table S1:** Primers for qPCR.

**Video S1:** cpr70218‐sup‐0002‐Video_S1.mp4.

## Data Availability

The data that support the findings of this study are available from the corresponding author upon reasonable request.
