# Supplementary material for: Light‐Inducible Activation of FGFR3 Facilitates Chondrocyte Maturation
Source: Cell Prolif. 2026 May 4;59(7):e70218. doi: 10.1111/cpr.70218 (PMC13325939; doi:10.1111/cpr.70218)
Supplement: Supplementary file 1 — Figure S1: Construction and validation of OptoFGFR3 cell line. (A) Confocal images of OptoFGFR3‐HEK293T cells before and after illumination with blue laser. Scale bar: 10 μm. (B) Confocal images of OptoFGFR3‐HEK293T cells after illumination with blue laser, counterstained with DAPI. Scale bar: 10 μm. (C) Immunostaining of pluripotency markers on OptoFGFR3 hPSCs. Scale bar: 200 μm. (D) Fluorescent image of OptoFGFR3 hPSCs after adding doxycycline. (E) qPCR analysis of marker genes expression of somite cells. (F) qPCR analysis of marker genes expression of sclerotome cells. (G) Immunostaining of type II collagen (COLII) and ACAN on chondrocyte pellets. Scale bar: 200 μm (left panel) and 100 μm (right panel). (H) Immunostaining of SOX9 and COLII on induced chondrocytes. Scale bar: 50 μm (left panel) and 100 μm (right panel). (I) Principal component analysis of RNA‐seq data from induced chondrocytes with or without blue light illumination. Figure S2: OptoFGFR3 inhibits the degeneration of human primary osteoarthritic chondrocytes. (A) Schematic illustration demonstrating the isolation of human primary chondrocytes and lentivirus transduction. (B) Principal component analysis of RNA‐seq data from primary chondrocytes with or without blue light illumination. (C) Gene‐set enrichment analysis (GSEA) on cell cycle‐related pathway. (D) GSEA on inflammation‐related pathway. (E) RNA‐seq expression levels of TGFB3, FN1 and FBN1. Table S1: Primers for qPCR. [file CPR-59-e70218-s002.docx]

Supporting Information for

**Light-inducible activation of FGFR3 facilitates chondrocyte maturation**

Mengze Sun^1,#^, Yun Zhao^2,#^, Yuqing Du^1^, Yifei Fan^1^, Kai Wang^2,^*, Xiaoqing Hu^1,^*

^1^ Department of Sports Medicine, Institute of Sports Medicine of Peking University, Beijing Key Laboratory of Sports Injuries, Peking University Third Hospital, Beijing 100191, China

^2^ Department of Physiology and Pathophysiology, School of Basic Medical Sciences, State Key Laboratory of Vascular Homeostasis and Remodeling, Beijing Advanced Center of Cellular Homeostasis and Aging-Related Diseases, Clinical Stem Cell Research Center, Peking University Third Hospital, Peking University, Beijing, 100191, China

*Correspondence: Xiaoqing Hu (huxiaoqingbd01@sina.com), Kai Wang ([kai.wang88@pku.edu.cn](mailto:kai.wang88@pku.edu.cn))

^#^ These authors contributed equally

**Table of Contents**

Materials and Methods

Supplementary Figures

Supplementary Table

OptoFGFR3 DNA sequence

Reference

**Materials and methods**

**Construction of PiggyBac-OptoFGFR3 and Lenti-OptoFGFR3 plasmid**

The DNA sequence encoding the Myr-tagged cytoplasmic domain of FGFR3 was synthesized commercially (Genewiz). The CRY2PHR-mCherry fragment was PCR-amplified from pCry2PHR-mCh-LRP6c (Addgene #42960). For the PiggyBac-OptoFGFR3 (PB-OptoFGFR3) construct, the two fragments were assembled into a doxycycline-inducible PiggyBac backbone carrying a puromycin selection cassette using Gibson assembly. For the lentiviral construct, the same fragments were inserted into an EF1α-driven lentiviral backbone using Gibson assembly.

**Cell culture**

The H9 human embryonic stem cell line (hESCs; WiCell, WA09) was maintained in eTeSR medium (STEMCELL Technologies, 100-1215) on plates pre-coated with hESC-qualified Matrigel (Corning, 354277). Cells were passaged every 3 days using 0.02% EDTA (Procell, PB180320) at a split ratio of 1:6, and replated in the presence of 5 μM Y27632 (Topscience, T1870) for the first 24 h. HEK293T cells (ATCC, CRL-3216) were cultured in Dulbecco’s Modified Eagle Medium (DMEM high glucose; Procell, PM150210) supplemented with 10% fetal bovine serum (FBS; ThermoFisher, A5256701) and 0.2% penicillin-streptomycin (P/S; Procell, PB180120). Medium was refreshed every 2-3 days and cells were passaged at 80–90% confluence using TrypLE Express (ThermoFisher, 12604021). Human primary chondrocytes were isolated from damaged regions of femoral condyles of total knee arthroplasty patients under the approval of the Human Ethics Committee of Peking University Third Hospital (approval No. 2013003), with informed consent obtained from each patient. Primary chondrocytes were cultured in Minimum Essential Medium α (MEM-α; Cytiva, SH30265.01) supplemented with 10% FBS and 1% P/S. All cells were maintained at 37°C in a humidified incubator with 5% CO_2_ and were routinely tested to ensure the absence of mycoplasma contamination.

**Establishment of PB-OptoFGFR3 HEK293T cell line**

HEK293T cells were transfected at 40–60% confluence. 9 μg PEI (Yeason, 40815ES) was diluted in 125 μL Opti-MEM (ThermoFisher, 11058021). 2 μg PB-OptoFGFR3 and 1 μg pEZY3-SPT encoding super PiggyBac transposase were diluted in 125 μL Opti-MEM. The PEI solution was added to the plasmid solution, mixed by vortexing, and incubated for 30 min at room temperature to allow complex formation. The resulting DNA/PEI complexes were added dropwise to one well of a six-well plate and incubated for 24 h. Transfected cells were then subjected to 5 μg/mL puromycin (Invivogen, ant-pr) selection for 7 days to establish a stable PB-OptoFGFR3 HEK293T cell line.

**Establishment of PB-OptoFGFR3 H9 hESC line**

2 μg PB-OptoFGFR3 and 1 μg pEZY3-SPT were electroporated into H9 hESCs using the Lonza Nucleofector 2b (Program: B-016). Selection was performed with 1 μg/ml puromycin for 3 days and clones were isolated for verification.

**Transduction of human primary chondrocytes with Lenti-OptoFGFR3**

Lentiviral particles encoding OptoFGFR3 were produced by transient transfection of HEK293T cells with the transfer plasmid and packaging plasmids using a standard protocol. Viral supernatants were collected 48 h post-transfection, filtered through a 0.45 μm filter, and concentrated. Human primary chondrocytes were seeded at an appropriate density and reached 40-60% confluency at the day of transduction. Lentiviral particles were added to the culture medium at a multiplicity of infection (MOI) of 10 in the presence of polybrene (8 μg/mL). After 24 h, the medium was replaced with fresh MEM-α medium. Transduced cells were cultured for 7 days before downstream assays to allow stable integration.

**Chondrogenic induction of the hESCs**

Differentiation of hESCs into induced chondrocyte pellets was performed as described previously with modifications [1–3]. Cells were dissociated into single cells using TrypLE Express and seeded at a density of 25,000 cells/cm^2^ on a Matrigel-coated plate. Basal differentiation medium (BDM) was prepared by adding 1× ITS-G (Procell, PB180429) and 1% P/S into DMEM/F12 medium (Corning, 10-092CVRC). After 24 h, differentiation was initiated using BDM supplemented with 3 μM CHIR99021 (Topscience, T2310) for the first 48 h. Medium was changed daily. For the next 48 h, medium was switched to BDM supplemented with 200 nM LDN193189 (Topscience, T6158) and 10 μM SB431542 (Topscience, T1726). Medium was changed daily. Then the cells in one well of a 6-well plate were dissociated using EDTA and seeded in two Matrigel-coated 10-cm plate in BDM supplemented with 20 ng/ml FGF2 (SinoBiological, 10014-HNAE) and 300 nM SAG (Topscience, T1779) for the next 48 h to generate sclerotome cells. Medium was changed daily. The sclerotome cells were dissociated into single cells using TrypLE Express, and 500,000 cells in 1 ml chondrogenic medium were pelleted at 300 g for 5 min in a 15-ml centrifuge tube. The chondrogenic medium was prepared by adding 1× ITS-G, 50 μg/ml vitamin C (Sigma, A8960), 40 μg/ml L-proline (Sigma, 81709), 0.9 mM sodium pyruvate (Procell, PB180422), 0.1 μM dexamethasone (Topscience, T1076), 10 ng/ml GDF5 (Novoprotein, CR36), 10 ng/ml TGF-β1 (SinoBiological, 10804-HNAC), 10 ng/ml BMP2 (SinoBiological, 10426-HNAE), 10 ng/ml TGF-β3 (SinoBiological, 10434-H01H) and 100 ng/ml FGF2 into DMEM high glucose medium (Procell, PM150210). Pellets were cultured in the same medium for 21 days. Cells were fed with fresh medium every other day. For dissociation of pellets into single cells, induced chondrocyte pellets were pooled and dissociated in 0.2% Collagenase II (ThermoFisher, 17101015) in DMEM/F12 for 2 h at 37°C.

**Live cell imaging and photoactivation of FGFR3**

OptoFGFR3-HEK293T cells were seeded in confocal dishes and imaged on a Nikon AXR confocal microscope. Photoactivation was performed using a 488‑nm laser delivered through a Galvano scanner, with the activation region adjusted via Nikon NIS-Elements AR software. For signaling pathway analysis by Western blot, cells in six-well plates were illuminated with 488‑nm blue light for 5 min, followed by immediate harvesting for protein extraction.

**Western Blot**

Total proteins were extracted using RIPA lysis buffer (Beyotime, P0013B) supplemented with protease and phosphatase inhibitors (Beyotime, P1045). Lysates were centrifuged at 12,000 g for 10 min at 4°C, and supernatants were collected. Protein concentration was determined by BCA assay (Beyotime, P0012). Samples were mixed with SDS loading buffer (Beyotime, P0015), boiled, and separated on 10% SDS–PAGE gels. Proteins were transferred onto PVDF membranes, blocked in 5% BSA in 0.1% TBST for 1 h at room temperature, and incubated with primary antibodies overnight at 4°C. After three washes with 0.1% TBST, membranes were incubated with HRP-conjugated secondary antibodies (goat anti-mouse 1:10000, ThermoFisher C31430100; goat anti-rabbit 1:5000, ThermoFisher C31460100) for 1 h at room temperature. Signals were developed using BeyoECL Plus (Beyotime, P0018S) and quantified with ImageJ. The primary antibodies used for Western Blot included mCherry (Abcam, ab183628, 1:1000), p-FGFR3 (Abcam, ab155960, 1:10000), p-ERK1/2 (CST, 4370T, 1:2000), ERK1/2 (CST, 4695S, 1:2000), α-tubulin (Abclonal, AC012, 1:1000), AKT (CST, 4691S, 1:1000), p-AKT (T308) (CST, 13038S, 1:1000), SOX9 (Abcam, ab185966, 1:1000), Collagen II (Proteintech, 28459-1-AP, 1:1000), Aggrecan (Abcam, ab3778, 1:1000), RUNX2 (Proteintech, 20700-1-AP, 1:1000), OGN (Proteintech, 12755-1-AP, 1:1000), GAPDH (Abclonal, A19056, 1:20000).

**Immunofluorescence**

Cells were washed with PBS, fixed with 4% paraformaldehyde (PFA) for 15 min at room temperature and permeabilized with 0.1% Triton-X 100 for 10 min. Then, cells were blocked in blocking buffer (PBS with 2% BSA) for 30 min and incubated with primary antibodies diluted in blocking buffer for 30 min at RT. Cells were then washed with PBS for three times and incubated with secondary antibodies diluted in blocking buffer as 1:500 for 15 min at RT. After three times wash with PBS, cells were counterstained with 4’,6-diamidino-2-phenylindole (DAPI, 0.5 μg/ml) for 10 min. Images were captured using Nikon AXR. The primary antibodies used for immunofluorescence include p-ERK1/2 (CST, 4370T, 1:200), Ki67 (Abcam, ab15580, 1:200), OCT4 (Santa Cruz, sc-5279, 1:200), NANOG (CST, 3580S, 1:800), SOX2 (CST, 3579S, 1:400), SOX9 (Abcam, ab185966, 1:200), type II collagen (Proteintech, 28459-1-AP, 1:200)

**Immunofluorescence on tissue sections**

Chondrocyte pellets were fixed with 4% PFA for 15 min at room temperature. Following fixation, samples were washed in PBS, dehydrated through a graded ethanol series, and embedded in paraffin. Paraffin blocks were sectioned at 4 μm thickness. For staining, sections were deparaffinized in xylene (2×10 min) and rehydrated through a descending ethanol series (100%, 95%, 80%, and 70%, 5 min each), followed by rinsing in distilled water. Antigen retrieval was performed by heating sections in EDTA buffer (pH 9.0) at 95°C for 20 min. Slides were then allowed to cool to room temperature and washed in PBS. Sections were blocked with blocking buffer (5% donkey serum in PBS) for 1 h at room temperature. Primary antibodies were diluted in blocking buffer and incubated overnight at 4°C in a humidified chamber. After three times wash with PBS, sections were incubated with secondary antibodies diluted in blocking buffer as 1:500 for 1 h at room temperature. Slides were washed and mounted using mounting medium with DAPI (Abcam, ab104139). Images were captured using Nikon AXR. The primary antibodies used here include type II collagen (Proteintech, 28459-1-AP, 1:200), aggrecan (Proteintech, 13880-1-AP, 1:200).

**RNA isolation and qPCR analysis**

Total RNA was extracted from hPSCs, somite cells and sclerotome cells using TRIzol reagent (ThermoFisher, 15596018CN). Purified total RNA was reverse transcribed using HiScript II 1st Strand cDNA Synthesis Kit (Vazyme, R211-01) following the manufacturer’s protocol. qPCR was performed using Taq Pro Universal SYBR qPCR Master Mix (Vazyme, Q712-02), and detection was achieved using Archimed X4 (RocGene). Expression of target genes was normalized to glyceraldehyde-3-phosphate dehydrogenase (GAPDH). All primer sequences for qPCR are included in Table S1.

**Cell viability assay (CCK-8)**Cell viability was assessed using the Cell Counting Kit-8 (Topscience, C0005) according to the manufacturer’s instructions. Briefly, induced chondrocytes were seeded in 96-well plates at a density of 5×10^3^ cells per well supplemented with 0.5 μg/ml doxycycline and allowed to adhere overnight. After 24 h, cells of experimental group were illuminated with 488‑nm blue light for 5 min. After another 72 h, 10 μL of CCK-8 solution was added to each well containing 100 μL of culture medium. Plates were incubated at 37°C for 4 h, and absorbance was measured at 450 nm using a microplate reader.

**RNA sequencing (RNA-seq)**

Total RNA was extracted using TRIzol reagent (ThermoFisher, 15596018CN). Libraries were prepared and sequenced by Annoroad. Briefly, 1 μg of total RNA was used for library preparation. The poly(A) mRNA was isolated using Oligo(dT) beads. The mRNA fragmentation was performed using divalent cations and high temperature. Priming was performed using Random Primers. First strand cDNA and the second-strand cDNA were synthesized. The purified double-stranded cDNA was then treated to repair both ends and add a dA-tailing in one reaction, followed by a T-A ligation to add adaptors to both ends. Size selection of Adaptor-ligated DNA was then performed using DNA Clean Beads. Each sample was then amplified by PCR using P5 and P7 primers and the PCR products were validated. Then libraries with different indices were multiplexed and loaded on an Illumina HiSeq instrument for sequencing using a 2×150 paired-end configuration according to the manufacturer’s instructions. For each group, three biological replicates were conducted.

**RNA-seq data analysis**

Quality control was performed using FastQC (v0.12.1). The reads were trimmed using TrimGalore (v0.6.10) to remove detected adapters. Then the reads were aligned to hg38 reference human genome with STAR (v2.7.11a) [4]. The expression count matrix of each sample was calculated using FeatureCounts (v2.0.6) [5]. The count matrix was normalized using DESeq2 (v1.44.0) for differential expression analyses [6]. Gene ontology (GO) analysis and gene-set enrichment analysis (GSEA) were performed with R package clusterProfiler (v4.12.6) and enrichplot (v1.24.4) [7]. Heatmaps were generated using pheatmap (v1.0.12) and ComplexHeatmap (v2.20.0).

**Statistical analysis**

Unless otherwise stated, data were expressed as means ± SEM of the mean. For comparisons between two groups, means were compared using unpaired two-tailed Student’s t-tests. No exclusion criteria were applied for all analyses. All statistical analyses were performed using GraphPad Prism 9 software (GraphPad Software Inc.). p < 0.05 was considered statistically significant.

**Supplementary Figures**


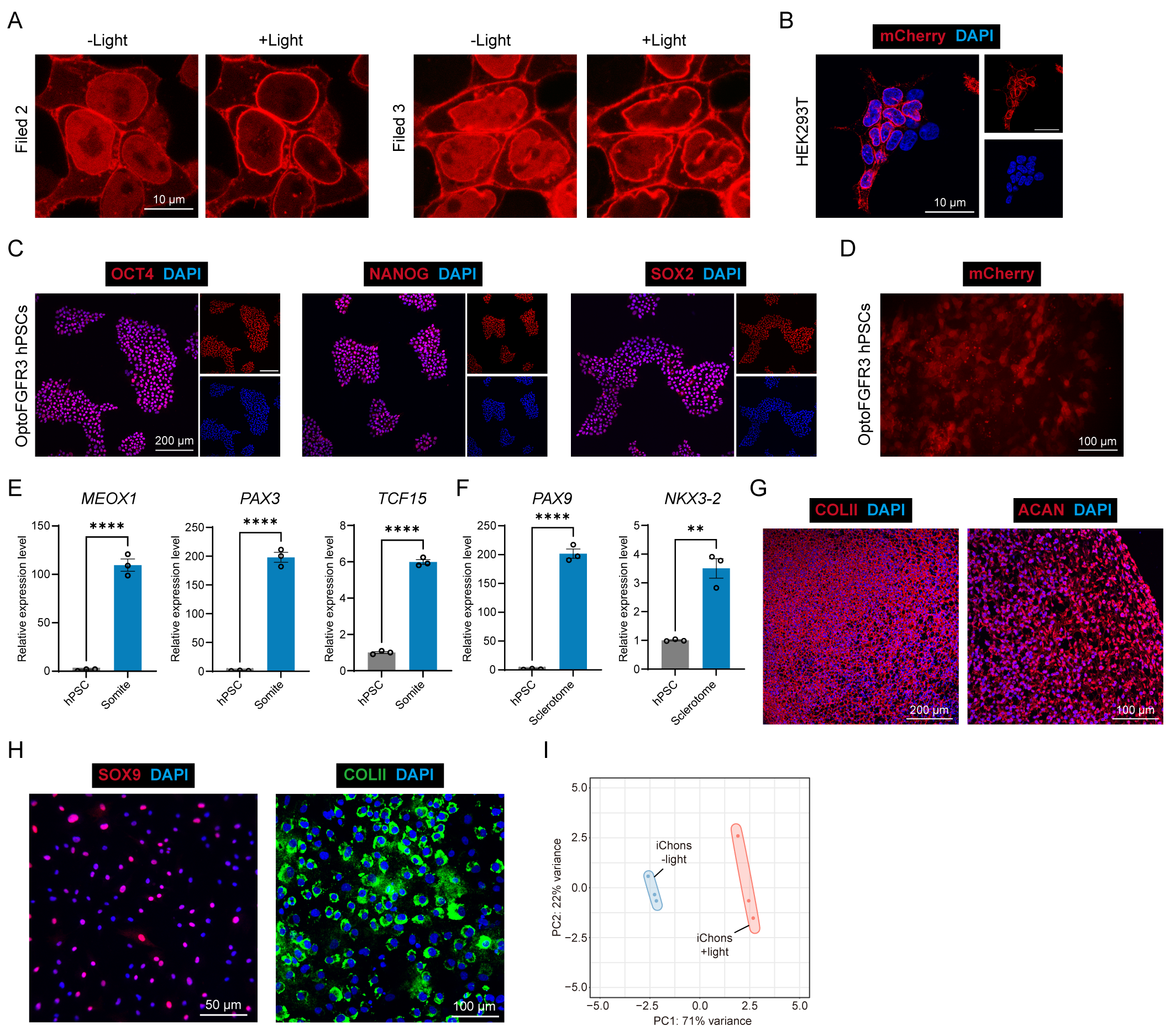


**Figure S1.** Construction and validation of OptoFGFR3 cell line. (A) Confocal images of OptoFGFR3-HEK293T cells before and after illumination with blue laser. Scale bar: 10 μm. (B) Confocal images of OptoFGFR3-HEK293T cells after illumination with blue laser, counterstained with DAPI. Scale bar: 10 μm. (C) Immunostaining of pluripotency markers on OptoFGFR3 hPSCs. Scale bar: 200 μm. (D) Fluorescent image of OptoFGFR3 hPSCs after adding doxycycline. (E) qPCR analysis of marker genes expression of somite cells. (F) qPCR analysis of marker genes expression of sclerotome cells. (G) Immunostaining of type II collagen (COLII) and ACAN on chondrocyte pellets. Scale bar: 200 μm (left panel) and 100 μm (right panel). (H) Immunostaining of SOX9 and COLII on induced chondrocytes. Scale bar: 50 μm (left panel) and 100 μm (right panel). (I) Principal component analysis of RNA-seq data from induced chondrocytes with or without blue light illumination.


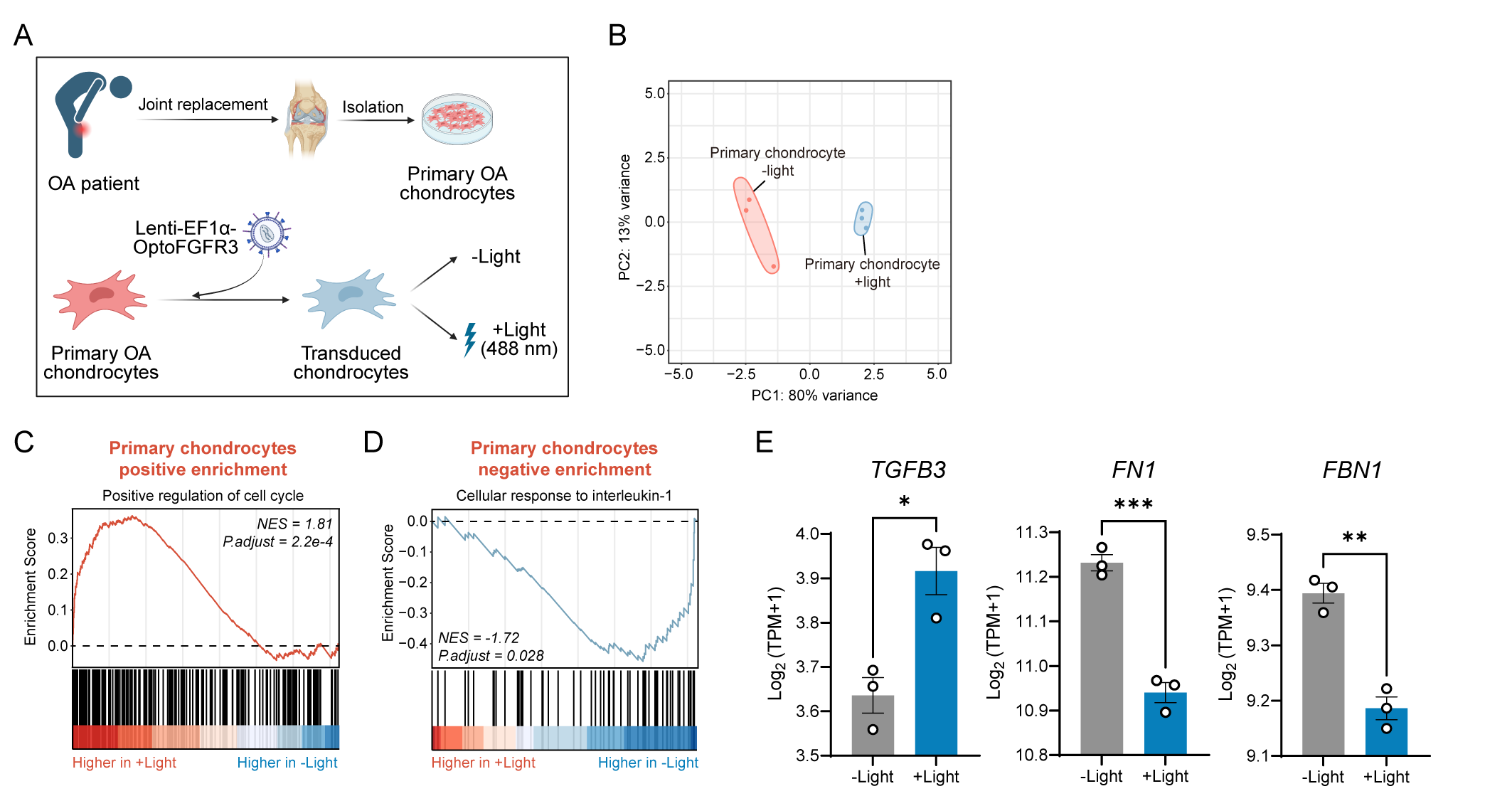


**Figure S2.** OptoFGFR3 inhibits the degeneration of human primary osteoarthritic chondrocytes. (A) Schematic illustration demonstrating the isolation of human primary chondrocytes and lentivirus transduction. (B) Principal component analysis of RNA-seq data from primary chondrocytes with or without blue light illumination. (C) Gene-set enrichment analysis (GSEA) on cell cycle-related pathway. (D) GSEA on inflammation-related pathway. (E) RNA-seq expression levels of *TGFB3*, *FN1* and *FBN1*.

**Supplementary Table**

**Table S1. Primers for qPCR**

| **Gene** | **Forward primer (5’-3’)** | **Reverse primer (5’-3’)** |
| --- | --- | --- |
| *MEOX1* | GGCAGCGTACCCTGACTTC | GGTCCCCATTTCCTTGGAACC |
| *PAX3* | AGCTCGGCGGTGTTTTTATCA | CTGCACAGGATCTTGGAGACG |
| *TCF15* | TTCTTTGTGTAGGGACCGGGG | GGGCAGGCTGAATGGATCCT |
| *PAX9* | AGCAGGGTCATTACGACTCAT | CTGGGGTACGAGTAGATGTGG |
| *NKX3-2* | TCCAGAACCGTCGCTACAAGA | CCGGGCAGGTATTGTCTCT |
| *GAPDH* | ACAACTTTGGTATCGTGGAAGG | GCCATCACGCCACAGTTTC |

**OptoFGFR3 DNA sequence**

atgggttcctccaaatccaagcccaaggacgcatctcagcgacgtcgcCGCCTGCGCAGCCCCCCCAAGAAAGGCCTGGGCTCCCCCACCGTGCACAAGATCTCCCGCTTCCCGCTCAAGCGACAGGTGTCCCTGGAGTCCAACGCGTCCATGAGCTCCAACACACCACTGGTGCGCATCGCAAGGCTGTCCTCAGGGGAGGGCCCCACGCTGGCCAATGTCTCCGAGCTCGAGCTGCCTGCCGACCCCAAATGGGAGCTGTCTCGGGCCCGGCTGACCCTGGGCAAGCCCCTTGGGGAGGGCTGCTTCGGCCAGGTGGTCATGGCGGAGGCCATCGGCATTGACAAGGACCGGGCCGCCAAGCCTGTCACCGTAGCCGTGAAGATGCTGAAAGACGATGCCACTGACAAGGACCTGTCGGACCTGGTGTCTGAGATGGAGATGATGAAGATGATCGGGAAACACAAAAACATCATCAACCTGCTGGGCGCCTGCACGCAGGGCGGGCCCCTGTACGTGCTGGTGGAGTACGCGGCCAAGGGTAACCTGCGGGAGTTTCTGCGGGCGCGGCGGCCCCCGGGCCTGGACTACTCCTTCGACACCTGCAAGCCGCCCGAGGAGCAGCTCACCTTCAAGGACCTGGTGTCCTGTGCCTACCAGGTGGCCCGGGGCATGGAGTACTTGGCCTCCCAGAAGTGCATCCACAGGGACCTGGCTGCCCGCAATGTGCTGGTGACCGAGGACAACGTGATGAAGATCGCAGACTTCGGGCTGGCCCGGGACGTGCACAACCTCGACTACTACAAGAAGACGACCAACGGCCGGCTGCCCGTGAAGTGGATGGCGCCTGAGGCCTTGTTTGACCGAGTCTACACTCACCAGAGTGACGTCTGGTCCTTTGGGGTCCTGCTCTGGGAGATCTTCACGCTGGGGGGCTCCCCGTACCCCGGCATCCCTGTGGAGGAGCTCTTCAAGCTGCTGAAGGAGGGCCACCGCATGGACAAGCCCGCCAACTGCACACACGACCTGTACATGATCATGCGGGAGTGCTGGCATGCCGCGCCCTCCCAGAGGCCCACCTTCAAGCAGCTGGTGGAGGACCTGGACCGTGTCCTTACCGTGACGTCCACCGACGAGTACCTGGACCTGTCGGCGCCTTTCGAGCAGTACTCCCCGGGTGGCCAGGACACCCCCAGCTCCAGCTCCTCAGGGGACGACTCCGTGTTTGCCCACGACCTGCTGCCCCCGGCCCCACCCAGCAGTGGGGGCTCGCGGACGgatggtggaggaggtagtggaggaggaggatcaatgaagatggacaaaaagactatagtttggtttagaagagacctaaggattgaggataatcctgcattagcagcagctgctcacgaaggatctgtttttcctgtcttcatttggtgtcctgaagaagaaggacagttttatcctggaagagcttcaagatggtggatgaaacaatcacttgctcacttatctcaatccttgaaggctcttggatctgacctcactttaatcaaaacccacaacacgatttcagcgatcttggattgtatccgcgttaccggtgctacaaaagtcgtctttaaccacctctatgatcctgtttcgttagttcgggaccataccgtaaaggagaagctggtggaacgtgggatctctgtgcaaagctacaatggagatctattgtatgaaccgtgggagatatactgcgaaaagggcaaaccttttacgagtttcaattcttactggaagaaatgcttagatatgtcgattgaatccgttatgcttcctcctccttggcggttgatgccaataactgcagcggctgaagcgatttgggcgtgttcgattgaagaactagggctggagaatgaggccgagaaaccgagcaatgcgttgttaactagagcttggtctccaggatggagcaatgctgataagttactaaatgagttcatcgagaagcagttgatagattatgcaaagaacagcaagaaagttgttgggaattctacttcactactttctccgtatctccatttcggggaaataagcgtcagacacgttttccagtgtgcccggatgaaacaaattatatgggcaagagataagaacagtgaaggagaagaaagtgcagatctttttcttaggggaatcggtttaagagagtattctcggtatatatgtttcaacttcccgtttactcacgagcaatcgttgttgagtcatcttcggtttttcccttgggatgctgatgttgataagttcaaggcctggagacaaggcaggaccggttatccgttggtggatgccggaatgagagagctttgggctaccggatggatgcataacagaataagagtgattgtttcaagctttgctgtgaagtttcttctccttccatggaaatggggaatgaagtatttctgggatacacttttggatgctgatttggaatgtgacatccttggctggcagtatatctctgggagtatccccgatggccacgagcttgatcgcttggacaatcccgcgttacaaggcgccaaatatgacccagaaggtgagtacataaggcaatggcttcccgagcttgcgagattgccaactgaatggatccatcatccatgggacgctcctttaaccgtactcaaagcttctggtgtggaactcggaacaaactatgcgaaacccattgtagacatcgacacagctcgtgagctactagctaaagctatttcaagaacccgtgaagcacagatcatgatcggagcagcagcccgggatccaccggtcgccaccatggtgagcaagggcgaggaggataacatggccatcatcaaggagttcatgcgcttcaaggtgcacatggagggctccgtgaacggccacgagttcgagatcgagggcgagggcgagggccgcccctacgagggcacccagaccgccaagctgaaggtgaccaagggtggccccctgcccttcgcctgggacatcctgtcccctcagttcatgtacggctccaaggcctacgtgaagcaccccgccgacatccccgactacttgaagctgtccttccccgagggcttcaagtgggagcgcgtgatgaacttcgaggacggcggcgtggtgaccgtgacccaggactcctccctgcaggacggcgagttcatctacaaggtgaagctgcgcggcaccaacttcccctccgacggccccgtaatgcagaagaagaccatgggctgggaggcctcctccgagcggatgtaccccgaggacggcgccctgaagggcgagatcaagcagaggctgaagctgaaggacggcggccactacgacgctgaggtcaagaccacctacaaggccaagaagcccgtgcagctgcccggcgcctacaacgtcaacatcaagttggacatcacctcccacaacgaggactacaccatcgtggaacagtacgaacgcgccgagggccgccactccaccggcggcatggacgagctgtacTAA

Green: Myr

Purple: FGFR3c

Blue: CRY2PHR

Red: mCherry

**Reference**

1. Lee, M.-S.; Stebbins, M.J.; Jiao, H.; Huang, H.-C.; Leiferman, E.M.; Walczak, B.E.; Palecek, S.P.; Shusta, E.V.; Li, W.-J. Comparative Evaluation of Isogenic Mesodermal and Ectomesodermal Chondrocytes from Human iPSCs for Cartilage Regeneration. *Sci Adv* **2021**, *7*, eabf0907, doi:10.1126/sciadv.abf0907.

2. Loh, K.M.; Chen, A.; Koh, P.W.; Deng, T.Z.; Sinha, R.; Tsai, J.M.; Barkal, A.A.; Shen, K.Y.; Jain, R.; Morganti, R.M.; et al. Mapping the Pairwise Choices Leading from Pluripotency to Human Bone, Heart, and Other Mesoderm Cell Types. *Cell* **2016**, *166*, 451–467, doi:10.1016/j.cell.2016.06.011.

3. Xi, H.; Fujiwara, W.; Gonzalez, K.; Jan, M.; Liebscher, S.; Handel, B.V.; Schenke-Layland, K.; Pyle, A.D. In Vivo Human Somitogenesis Guides Somite Development from hPSCs. *Cell Reports* **2017**, *18*, 1573–1585, doi:10.1016/j.celrep.2017.01.040.

4. Dobin, A.; Davis, C.A.; Schlesinger, F.; Drenkow, J.; Zaleski, C.; Jha, S.; Batut, P.; Chaisson, M.; Gingeras, T.R. STAR: Ultrafast Universal RNA-Seq Aligner. *Bioinformatics* **2013**, *29*, 15–21, doi:10.1093/bioinformatics/bts635.

5. Liao, Y.; Smyth, G.K.; Shi, W. featureCounts: An Efficient General Purpose Program for Assigning Sequence Reads to Genomic Features. *Bioinformatics* **2014**, *30*, 923–930, doi:10.1093/bioinformatics/btt656.

6. Love, M.I.; Huber, W.; Anders, S. Moderated Estimation of Fold Change and Dispersion for RNA-Seq Data with DESeq2. *Genome Biol.* **2014**, *15*, 550, doi:10.1186/s13059-014-0550-8.

7. Wu, T.; Hu, E.; Xu, S.; Chen, M.; Guo, P.; Dai, Z.; Feng, T.; Zhou, L.; Tang, W.; Zhan, L.; et al. clusterProfiler 4.0: A Universal Enrichment Tool for Interpreting Omics Data. *Innov.* **2021**, *2*, 100141, doi:10.1016/j.xinn.2021.100141.
